# The Vertical Rectus Abdominis Musculocutaneous Flap As a Versatile and Viable Option for Perineal Reconstruction

**Published:** 2017-01-16

**Authors:** Demetrius M. Coombs, Nirav B. Patel, Matthew R. Zeiderman, Michael S. Wong

**Affiliations:** ^a^Drexel University College of Medicine, Philadelphia, Pa; ^b^Division of Plastic & Reconstructive Surgery, University of California, Davis, Sacramento, Ca

**Keywords:** perineal reconstruction, VRAM, squamous cell carcinoma, radiation-induced tissue damage, wound healing

## DESCRIPTION

A 55-year-old man with a history of squamous cell carcinoma of the anus status-post radiation presented with an ulcerated lesion warranting pelvic exenteration and abdominoperineal resection (APR) and leaving a significant soft-tissue defect involving the right perineum. A right vertical rectus abdominis musculocutaneous (VRAM) flap was selected for reconstruction.

## QUESTIONS

1. **What reconstructive options exist in the plastic surgeon's arsenal that address defects of the perineum? In brief, what are the advantages of each?**
2. **When planning a procedure for perineal reconstruction, what are the indications for harvesting a VRAM flap?**
3. **In brief, what is the effect of radiation-induced tissue damage on reconstructive surgical outcomes and, ultimately, on wound healing?**
4. **What postoperative complications are most typically associated with perineal reconstruction and use of a VRAM flap? What strategies exist to mitigate these complications?**

## DISCUSSION

Successful perineal reconstruction provides wound coverage, facilitates healing, employs vascularized tissue with sufficient bulk, maintains urogenital and anorectal function,^1^^,^^2^ and helps sustain upright posture and ambulation.^1^ Genitourinary and digestive tract malignancies result in large resection defects and provide impetus for complex perineal reconstruction. Locoregional flaps are preferred when direct closure is not feasible.^1^^,^^2^ A VRAM flap is ideal where bulk and a large skin paddle are required, for example, pelvic floor, perineal skin, and posterior vaginal wall damage. A tubularized VRAM flap can provide a neovagina.^1^^,^^2^ The gracilis flap repairs rectovaginal and rectourethral fistulas, restores continence following anal sphincter damage, and creates a neovagina following pelvic exenteration.^1^ The pudendal, or modified Singapore flap, is partially sensate and reliable for postoperative sexual function. While suitable for smaller defects to the anterior or lateral vaginal wall, it remains susceptible to radiation injury. Despite relatively high donor-site morbidity, thigh flaps cover smaller defects and may be sensate.^1^ Additional options include the gluteus maximus, omental, and tensor fascia lata. Ileum, cecum, and sigmoid have been described for vaginal reconstruction but are complication- prone.^2^

**Figure 1:**
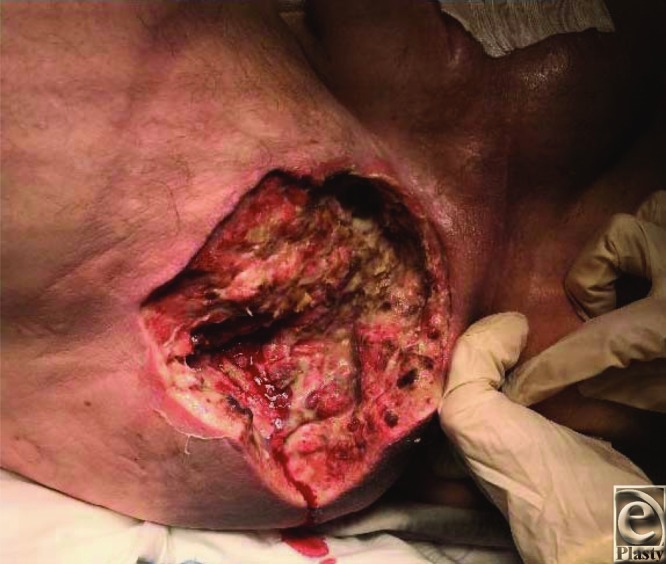
Ulcerated right thigh lesion and persistent necrotizing soft-tissue infection upon presentation, despite multiple previous debridements (the patient in lithotomy).

**Figure 2:**
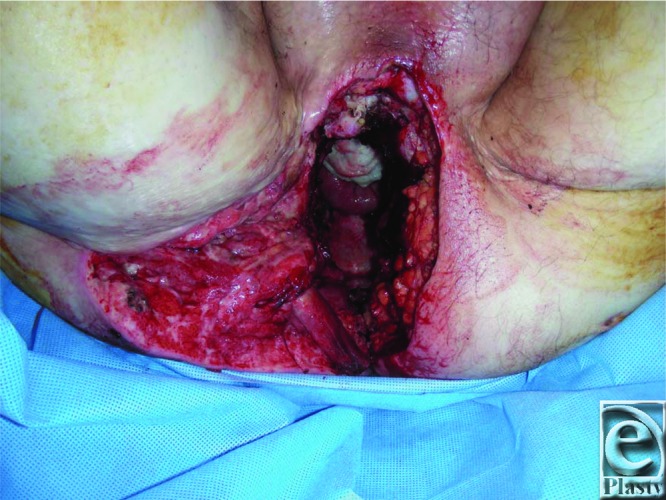
Significant soft-tissue defect involving the right perineum and gluteal cleft following pelvic exenteration and abdominoperineal resection (in lithotomy; the top of photograph represents the patient's anterior perineum).

**Figure 3:**
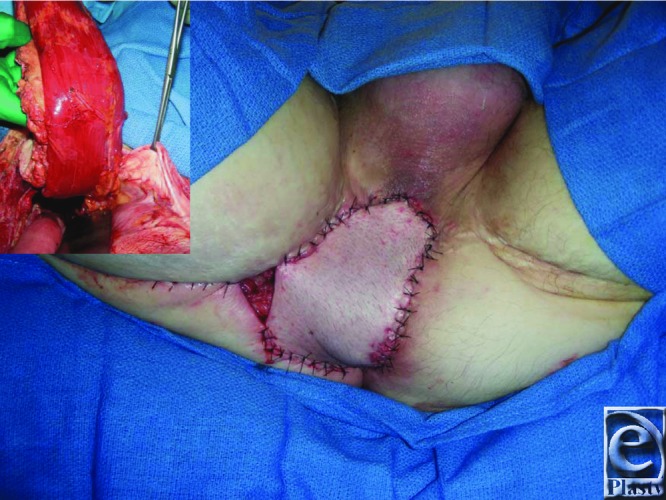
Immediately following right vertical rectus abdominis musculocutaneous flap inset and final closure (the patient in lithotomy; smaller photograph demonstrates elevation of the flap and the pedicle prior to rotation, inset, and closure).

The VRAM flap is a Mathes and Nahai type III flap with dual perfusion from the deep superior and inferior epigastric arteries.^3^ The patient described presented a significant defect at a previously irradiated right perineum/gluteal cleft and after pelvic exenteration and APR requiring midline laparotomy (Figs 1 and 2). An inferiorly based, pedicled VRAM flap provided sufficient bulk (7 x 30-cm skin paddle) and allowed primary abdominal closure and placement of a stoma in the contralateral rectus^1^^,^^2^ (Fig 3). Küntscher et al^3^ demonstrated that inferiorly pedicled VRAM flaps remain safe reconstructions for groin, hip, and perineum defects, even in higher risk settings of peripheral vascular disease, radiation, and osteomyelitis. Nelson and Butler^4^ reported fewer complications with VRAM flap versus thigh flap, without increased abdominal morbidity, following immediate reconstruction of pelvic exenteration and APR defects.

Radiation exposure impairs cellular wound healing mechanisms, continuing cellular regeneration with prolonged inflammation.^5^ Manifestations include cellular matrix accumulation, decreased wound strength, poor soft-tissue reconstitution, and erratic collagen bundle formation.^5^ Consequences include wound dehiscence, infection, sinus tracts and fistulas, contractures, hyperpigmentation, scarring, fibrosis, skin atrophy, desquamation, ulceration, and vessel rupture.^1^^,^^2^^,^^5^ Where 81% of patients received chemoradiotherapy perioperatively, Lefevre et al^6^ demonstrated that VRAM flaps decrease wound complications, perineal closure delay, and healing time following APR for anal carcinoma.

Wound complications occur in 25% to 60% of patients with prior chemoradiotherapy who undergo APR.^6^ VRAM-associated complications include fluid collection, maceration, bulge/hernia, evisceration, flap loss secondary to ischemia and tissue necrosis, and reoperation.^1^^,^^2^^,^^7^ Reategui et al^8^ reported that VRAM flap reconstruction in patients who undergo APR was associated with earlier onset of “phantom rectum,” that is, sensation of an intact, functioning rectum. Campbell and Butler^7^ proposed modifications to the traditional VRAM flap that improved outcomes in patients with previously irradiated APR and pelvic exenteration, including sparing fascia, using mesh inlay, combining omentum, and using an “extended” VRAM flap.

**Figure 4:**
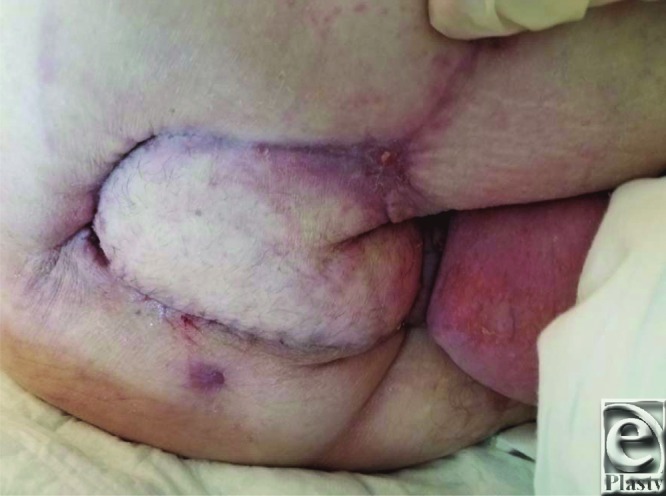
Approximately 2.5 months postoperatively (note the evidence of wound healing delay evident near the posterior region of the flap). The right anterolateral perineum, a previously irradiated site unable to be approximated during the reconstruction, has contracted and granulated to the point of reepithelialization (the patient in the left lateral decubitus position; right side of the photograph demonstrates the patient's anterior perineum).

Perineal soft-tissue defects provide plastic surgeons with unique reconstructive challenges, mandating consideration of genitalia and patient expectations to optimize psychosocial well-being. A multitude of options respect the reconstructive ladder concept and address perineal defects. Flap selection remains surgeon-dependent, combined with locoregional tissue requirements and availability. Radiation exposure imparts deleterious postoperative and wound healing complications. Reliable tissue coverage of the described patient's pelvic wound was achieved with a unipedicled VRAM flap due to an inherently large arc of rotation, robust blood supply, and ample tissue bulk (Fig 3). Prior irradiation contributed to a residual 2.5 x 3 cm wound on the right medial thigh, even after flap inset and local tissue rearrangement; nonetheless, the patient healed well with negative pressure wound therapy (Fig 4).
